# Editorial: Risk and protective factors, family environment and (a)typical neurodevelopmental outcomes

**DOI:** 10.3389/fpsyg.2023.1221338

**Published:** 2023-06-20

**Authors:** Livio Provenzi, Giorgia Bussu, Valentina Riva

**Affiliations:** ^1^Department of Brain and Behavioral Sciences, University of Pavia, Pavia, Italy; ^2^Developmental Psychobiology Lab, IRCCS Mondino Foundation, Pavia, Italy; ^3^Development and Neurodiversity Lab, Developmental Psychology Division, Department of Psychology, Uppsala University, Uppsala, Sweden; ^4^Child Psychopathology Unit, Scientific Institute IRCCS E. Medea, Lecco, Italy

**Keywords:** neurodevelopment, autism, ADHD, epigenetics, parenting, siblings

Child development is a non-linear, chaotic process that can be observed across different levels of analysis, each one being only partially predictable and as a whole concerning an open, interacting system that cannot be reduced to simplistic observations of isolated processes concerning the individual child alone (Sander, 2000; Smith and Thelen, 2003). Rather, child development arises from the continuous interaction between genetic predispositions and environmental conditions, and as part of a broader ecological system that spans from immediate family environment, to larger communities, society and culture, each one influencing a child's daily life experience and development more in general (Bronfenbrenner and Morris, 2006).

As humans, brain maturation begins in the pre-natal stage and continues after birth, when in the early postnatal life—especially during the first thousand days after conception—we assist to an incredible sprout of neuroplasticity which interacts with environmental exposures to shape the emerging behavior or function (Berretta et al., 2021; Scher, 2022). Developmental research on early infancy has shown how the optimal environmental conditions for positive and adaptive growth and development include the presence of a sensitive and responsive caregiving context in which the infant finds contingent responses to their needs and appropriate care (Linnér and Almgren, 2020; Wilder and Semendeferi, 2022). At the same time, from a developmental neuro-constructivist perspective, it is widely accepted that even tiny asynchronies or mismatches between genetic predispositions and environmental features that might occur early in life can have relevant cascading consequences for the emergent phenotypic outcomes in typical and atypical development (Karmiloff-Smith, 1998, 2006).

Early environmental influences appear then fundamental to carve out the developmental landscape established by genetic predispositions and shape a child's developmental trajectory. These interacting influences are dynamic in nature and lead to a large variability among individual infants and across development. In this context, small individual differences early in development can be compensated for by alternative, adaptive pathways leading to a normative developmental outcome, or amplified over developmental cascades leading to more neurodivergent phenotypes, like autism or ADHD (D'Souza and Karmiloff-Smith, 2016). Consistently, the environment in which child development is encapsulated is capable of affecting the programming of fetal and postnatal growth processes and it does so mainly through epigenetic mechanisms that are especially sensitive to variations in the quality of caregiving exposures (Provenzi et al., 2020; Unternaehrer et al., 2021). Notably, the consequences for cognitive, behavioral and emotional child development are relevant whether variations in the quality of the environment include traumatic or stressful conditions (Devlin et al., 2010; Barker et al., 2020) or nurturing and sensitive parenting (Murgatroyd et al., 2015; Unternaehrer et al., 2015). In sum, the emerging behavioral epigenetic field provides the neuro-constructivist paradigm with specific biochemical mechanisms that further support the notion by which it is at the interface between genes and environment that developmental trajectories are shaped.

Consistent with this view of human development, we have launched the present Research Topic in 2022 to collect evidence of the role of the care environment in defining elements of risk and protection for children with typical development and for children who show neurodivergent conditions. The Research Topic is now published and includes six different papers written by colleagues/samples from Europe (Poland, Spain and Italy), America (Guatemala), Africa (Egypt), and Asia (Saudi Arabia and China). Here, we summarize their contributions highlighting the implications for the advancement of our understanding of the role of the family and caregiving environment in shaping typical and atypical developmental trajectories in children.

The role of family environment in the autistic condition, with all its complexity and different components, is analyzed by two contributions of this Research Topic. Dong et al. addressed the role of environmental predictors in a sample of autistic children. In a large cohort study, sociodemographic, individual, and social factors were linked with developmental quotient in autistic children. They found that among the investigated factors, lower Vitamin D concentration, the severity of autistic traits, and a poor parent-child interaction played a significant role on cognitive development. On the other hand, maternal and paternal educational level, household income, and screen time exposure did not affect cognitive skills. In their contribution, Sipowicz et al. focused on loneliness and depression traits in adult siblings of autistic individuals, compared to siblings of neurotypical individuals and individuals without siblings. The authors found significant group differences, and having an autistic sibling increased depression and loneliness levels. In addition, women showed higher levels of loneliness and depression traits compared to men. The authors highlight the importance and need of screening for depression within families of autistic individuals.

Two additional studies investigated environmental influences on child development in relation to two known areas of relevant concern: screen use and diet. Zoromba et al. reported on the link between behavioral problems among preschool children and media exposure in Egypt. Capitalizing from a large cohort of children, the authors documented a daily media exposure above recommended time for this age group (100 min), with longer durations of exposure being significantly correlated with specific behavioral problems—including hyperactivity, conduct problem, and anxiety. While this linear association should not suggest a simplistic and causal interpretation, this study contributes to the existent literature on negative correlates of screen use in childhood and the need to concurrently engage children into alternative social activities by reporting data from underrepresented populations, a critical element for further cross-cultural comparisons and investigations. Company-Cordoba et al. conducted a study on how food insecurity and household food consumption might impact the cognitive performance of children at risk of social exclusion. The study was conducted in Guatemala where children diet is further challenged by specific hazards related to the socioeconomic difficulties. The authors showed that despite rural and urban groups did not differ in terms of food insecurity, when considering rural areas only, differences were found between groups with food security and insecurity in attention and executive function tasks. More specifically, protein food consumption (e.g., meat and fish) was a relevant factor in executive performance. These findings should inform policy-makers on the implementation of initiatives to ensure food security in families at risk of social exclusion and therefore supporting a more sustainable and balanced diet across the population.

Two studies focused on the role of caregiving environment in samples of children with visual disabilities. Gui et al. conducted an observational study focusing on the quality of parent-child interaction in two different groups of children: one with total blindness (TB) and one with partial blindness (PB). They found that parents of TB children had higher parenting stress and lower perceived social support scores than parents of PB children. While there was no difference in the time TB and PB children spent displaying joint engagement behaviors during parent-child interaction, TB children directed their gaze and face less often toward their parents than PB children. A trend for an association between this behavior and maternal stress was also highlighted, suggesting the opportunity to invest in early interventions that support parental caregiving for this specific population. Notably, Provenzi et al. reported on a single case study of a parent-child intervention conducted with a child with severe visual impairment and his blind father. The study reports on the session-by-session improvements that were noted in this dyad not only in terms of an increase and appropriateness of child communicative signals, but also in terms of a general change of the interactive pattern shown by the dyad. By using an observational grid to code the videotaped sessions, the authors were able to provide a view of therapeutic change inspired by the dynamic system theory and it was possible to describe a movement of the dyad as a whole toward a more reciprocally satisfactory interaction.

In sum, this Research Topic provides evidence on the influence of the family and caregiving environment on child development, highlighting its relevant role in the presence of specific environmental or genetic risk factors, and further highlighting how improving the quality of the early care environment might be crucial in supporting the health and development of children with neurodevelopmental conditions. By accumulating such evidence, our hope is that a virtuous connection among researchers, clinicians and policy-makers might increase our possibility to protect and promote child development by investing in sensitive caregiving and parental support.

## Author contributions

All authors listed have made a substantial, direct, and intellectual contribution to the work and approved it for publication.
